# Is There Any Non-functional Training? A Conceptual Review

**DOI:** 10.3389/fspor.2021.803366

**Published:** 2022-01-13

**Authors:** Bernardo N. Ide, Amanda P. Silvatti, Moacir Marocolo, Clarcson P. C. Santos, Bruno V. C. Silva, Dustin J. Oranchuk, Gustavo R. Mota

**Affiliations:** ^1^Exercise Science, Health and Human Performance Research Group, Department of Sport Sciences, Institute of Health Sciences, Federal University of Triângulo Mineiro, Uberaba, Brazil; ^2^Department of Physical Education, Federal University of Viçosa, Viçosa, Brazil; ^3^Physiology and Human Performance Research Group, Department of Physiology, Federal University of Juiz de Fora, Juiz de Fora, Brazil; ^4^Research Group on Metabolic Diseases, Physical Exercise and Health Technologies, Bahiana School of Medicine and Public Health, Salvador, Brazil; ^5^Department of Sport Sciences, University Center of Belo Horizonte, Belo Horizonte, Brazil; ^6^Acumen Sport and Shoulder Clinic, Calgary, AB, Canada

**Keywords:** core training, exercises, flexibility, fitness, periodization

## Abstract

This conceptual review investigates whether *functional training* (FT) is a different approach from traditional strength, power, flexibility, and endurance (aerobic or cardiorespiratory) training already adopted in the physical training plan of professional, recreational athletes, healthy, and older adults. The 20 most recent papers published involving FT were searched in the PubMed/Medline database. Definition, concepts, benefits, and the exercises employed in FT programs were analyzed. The main results were: (a) there is no agreement about a universal definition for FT; (b) FT programs aim at developing the same benefits already induced by traditional training programs; (c) exercises employed are also the same. The inability to define FT makes the differentiation from traditional training programs difficult. Physical training programs can be easily described and classified as strength, power, flexibility, endurance, and the specific exercises employed (e.g., traditional resistance training, ballistic exercises, plyometrics and Olympic-style weightlifting, continuous and high-intensity interval training). This apt description and classification may provide consistent and clear communication between students, coaches, athletes, and sports scientists. Based on the current evidence and to avoid confusion and misconceptions, we recommend that the terms FT, high-intensity FT, and functional fitness training no longer describe any physical training program.

## Introduction

Strength, power, endurance and flexibility are well-defined concepts within exercise prescription and muscle performance (Cormie et al., 2011a; Granata et al., 2018; Nuzzo, 2020), nutritional requirements (Baar, 2014) and the study of specific neuromuscular, cardiovascular, and metabolic adaptations (Cormie et al., 2011b; Egan and Zierath, 2013; Granata et al., 2018). Strength and power training encompass short-duration activities performed at high- or near maximal intensities, increasing the capacity to perform high-force, and high-velocity efforts (Nader, 2006; Cormie et al., 2011b). Exercises employed in these programs involve traditional resistance training, ballistic exercises, plyometrics and Olympic-style weightlifting (Cormie et al., 2011a). On the other hand, endurance training (aerobic or cardiorespiratory) encompasses activities performed at various intensities, lasting for several minutes up to hours (Åstrand, 2000), increasing the capacity to sustain repetitive high and low-intensity efforts (Granata et al., 2018), encompassing the application of both continuous and high-intensity interval training (HIIT) (Buchheit and Laursen, 2013; Granata et al., 2018).

Otherwise, despite these well-consolidated characterizations of sports physical demand, training programs designs and adaptations, an increasing number of articles introduce apparently “new” physical training programs. Nowadays, it is common to hear from students, coaches, and athletes: “*I'm working with functional training (FT),” “I'm engaged on a high-intensity FT program,” “I'm investigating the neuromuscular responses to functional training.”* These statements caught our attention and have been previously criticized (Wirth et al., 2017; Ide et al., 2021). Recently we have raised issues regarding the concepts and definition of FT (Ide et al., 2021). Unfortunately, we found inconsistencies and misconceptions on the FT definition, cited references that do not support the statements, and no differences regarding benefits and training methods already used in sports training (Ide et al., 2021).

The dissemination of inconsistent and inaccurate concepts and definitions can induce irreparable professional conduct. Therefore, the present conceptual review aimed to investigate whether FT programs are different from strength, power, flexibility, and endurance training programs already adopted in the physical training of professional and recreational athletes, healthy adults, and geriatric populations. Based on our recent article about the inconsistencies in the concepts and characteristics of FT (Ide et al., 2021), we hypothesized that FT has no universal definition and that FT programs aim to induce the same neuromuscular adaptations as strength, power, flexibility, and endurance training programs. FT exercises are also already employed in athletes' training programs.

## Methods

To attend to the purposes of the conceptual review study, we performed a non-exhaustive search for the 20 most recent papers published about FT present in PubMed/Medline database. During the study design and initial search, we learned that, in 2020, some researchers on the topic (i.e., functional training) published a conceptual update (Silva-Grigoletto et al., 2020). Therefore, our criteria for choosing the twenty most recent papers aimed at considering the latest updates to the concept (i.e., functional training). Bearing this in mind and making our article unique, we felt unnecessary and outdated to perform a review going behind 2020 (i.e., older papers). The formal search was completed in April 2021. The article's reference list was consulted for additional definitions. The heterogeneity of the studies was considerable (e.g., exercise protocols, fitness level of the participants, variables measured). Thus, we have decided not to evaluate the studies from a statistical point of view. Instead, we performed a qualitative analysis, focusing on the FT definitions, exercises employed, and neuromuscular adaptations reported by the authors. All co-authors read this qualitative analysis carefully, and edits have been incorporated.

For this study, the term *traditional training programs* was considered to reference strength, power, flexibility, and endurance training (aerobic or cardiorespiratory) already adopted as a part of physical training plan of professional, recreational athletes, healthy, and older adults. Strength and power exercises used in these programs are the traditional resistance training performed both in machines or with free weights, ballistic exercises, plyometrics, and Olympic-style weightlifting (Cormie et al., 2011a). Endurance training (aerobic or cardiorespiratory) exercises used in these programs encompass the application of both continuous and high-intensity interval training (HIIT) (Buchheit and Laursen, 2013; Granata et al., 2018).

## Results

Examining the search results, we found additional FT “variations” (e.g., high-intensity FT, and functional fitness) included in the analysis of definitions, neuromuscular adaptations, and exercises employed. In addition to the articles, three textbooks were included (Boyle, 2004, 2016; Fleck and Kraemer, 2014).

### Definitions of Functional Training

Tables 1–3 present the FT, high-intensity FT (HIFT), and functional fitness (FF) definitions, respectively.

**Table 1:** Definitions of functional training.

**References**	**Definition of functional training**
Lajoso-Silva et al. (2021)	FT utilizes multi-articular movements to improve balance, increase muscular power and strength, and enhance conditional and coordinative capacities.
Gali et al. (2021)	FT combines neuromuscular control, joint mobility and stability, central stability, trunk alignment and lower limb joint. Unlike traditional muscle strengthening programs, several joints and muscles are exercised in the three planes of movement during FT, simultaneously challenging the brain and the body.
McLaughlin et al. (2020)	Functional training uses functional activities as the training stimulus and is based on the theoretical concept of task specificity.
Farrokhian et al. (2020)	FT is a set of sports activities that are based on daily routine activities such as walking, climbing up stairs and going down, getting up and sitting down and move light things. FT was focused improving physical fitness such as endurance, strength, flexibility, and balance.
Da Silva-Grigoletto et al. (2020)	FT involves resistance training and associated techniques to develop strength, as well as balance, motor coordination, power, and muscle endurance, increasing the ability of individuals to execute ADL, whether they be simpler tasks of daily living or more complex athletic maneuvers.
Cheng et al. (2020)	A form of training that uses modular movements that involve the recruitment of multiple muscle groups, FT is the only program that combines weightlifting, gymnastics, and metabolic conditioning in one continuous session.
Peterson (2017)	FT is designed to enhance the ability of exercisers to meet the demands of performing a wide range of ADL at home, work, or play without undue risk of injury or fatigue.
Aragao-Santos et al. (2020)	FT is a multicomponent training method, which stimulates different physical capacities in the same session. This training method can be carried out with an emphasis on traditional exercises such as squats to improve the strength of lower limbs (element-based functional training) or using exercises more like daily activities such as carrying actions or sit and get up from the floor (task-specific-based functional training).
La Scala Teixeira et al. (2017)	The development of different physical capacities in an integrated and balanced manner to provide autonomy, efficiency and safety during activities related to ADLs, work and/or sports. For this purpose, FT uses strength exercises generally characterized by integrated, multi-joint/multi-segment, asymmetrical, multi-planes, acyclic, intermittent, speedy, and unstable movements that emphasize core stability.
Fleck and Kraemer (2014)	The training that is meant to increase performance in some type of functional task, such as activities of daily living or tests related to athletic performance. FT could refer to virtually any type of training meant to increase motor performance.
Boyle (2016)	Functional training on the other hand uses many concepts developed by sport coaches to train speed, strength, and power to improve sport performance and reduce incidence of injury.
Boyle (2004)	Functional training can therefore be described as purposeful training. In fact, functional training is more accurately represented as “sports-general” training. Functional training is a system that encourages the training of balance and the balancing of training. It is characterized by actions such as squatting and lunging or pushing and pulling. Functional training is best described as a continuum of exercises that teach athletes to handle their own body weight in all planes of movement.

*ADLs, activities of daily living; FT, functional training; HIIT, high-intensity interval training*.

**Table 2:** Definitions of high-intensity functional training.

**References**	**Definition of high-intensity functional training**
Feito et al. (2018)	A training style [or program] that incorporates a variety of functional movements, performed at high intensity [relative to an individual's ability], and designed to improve parameters of general physical fitness (e.g., cardiovascular endurance, strength, body composition, flexibility, etc.) and performance (e.g., agility, speed, power, strength, etc.).
Teixeira et al. (2020)	HIFT is a modality characterized by presenting high volumes and training intensities with constantly varied exercises with or without any recovery interval between the series. HIFT training sessions consist of Olympic weightlifting exercises (e.g., clean and jerk, snatch), gymnastics (e.g., lunges and pull-ups) and metabolic conditioning (e.g., running and rowing). In addition to the diversity of functional movements performed in high intensity, HIFT aims to improve physical conditioning variables (i.e., strength, body composition, among others) and performance (i.e., speed, power, among others).
Gomes et al. (2020)	Exercise regimen characterized by high intensity, constant variation, and functional movement is often performed in rapid, successive repetition with limited or no recovery time. HIFT is based on the concept of increased work capacity over time while using a variety of exercise modalities, including mono-structural (e.g., running, rowing, etc.), as well as body weight movements (e.g., squats, push-ups, etc.) and weightlifting derivatives (e.g., snatch, shoulder press, deadlift, etc.).
Browne et al. (2020)	HIFT incorporates many of the same principles as HIIT, including the relatively high work-to-rest intervals. However, HIFT training goes further and weaves multimodal resistance training with cardiovascular exercises. HIFT consists of a variable series of these functional whole-body exercises with little rest, while HIIT consists of unimodal, single-plane movements with distinct periods of low-intensity activity or rest.
Ben-Zeev et al. (2020)	HIFT is a form of physical activity that can be modified to any fitness level and elicits greater muscle recruitment than repetitive aerobic exercises, thereby improving cardiovascular endurance, strength, and flexibility. HIFT emphasizes functional, multi-joint movements via HIIT and muscle-strengthening exercises.

*ADLs, activities of daily living; HIFT, high-intensity functional training; HIIT, high-intensity interval training*.

**Table 3:** Definitions of functional fitness.

**References**	**Definition of functional fitness**
Thompson (2021) and Tibana et al. (2019)	A trend toward using strength training to improve balance, coordination, muscular strength, and endurance to improve activities of daily living typically for older adults and in clinical populations.
Tibana et al. (2019)	A relatively new form of exercise (also known as HIFT; extreme conditioning programs) that is currently being marketed to a wide range of active (athletes, military) and inactive populations. The competitive functional fitness (e.g., CrossFit®) often consists of a variety of training methods, such as weightlifting/powerlifting, repeated gym bodyweight exercises, cardiovascular exercises, sprints, and flexibility mixed to achieve a high global performance.
Peterson (2017)	Functional fitness is a by-product of the synergistic integration of the various components of fitness (physical and neuromuscular) and the muscle groups and joints involved in a movement activity or training effort.

*ADLs, activities of daily living; HIFT, high-intensity functional training; HIIT, high-intensity interval training*.

### Neuromuscular Adaptations to Functional Training

The neuromuscular adaptations proposed by each training program were extracted from the definitions and additional descriptions presented in the articles. Tables 4–6 present neuromuscular adaptations to FT, HIFT, and FF training programs.

**Table 4:** Neuromuscular adaptations provided by functional training programs.

**References**	**Neuromuscular adaptations to functional training**			
Lajoso-Silva et al. (2021)	Strength		Power		Balance		Conditional and coordinative capacities	
Gali et al. (2021)	Neuromuscular control, joint mobility and stability, central stability, trunk alignment and lower limb joint							
Farrokhian et al. (2020)	Strength		Balance		Endurance		Flexibility	
Da Silva-Grigoletto et al. (2020)	Strength		Power	Balance		Endurance		Coordination
Cheng et al. (2020)	Strength		Power	Balance		Endurance		Coordination
Peterson (2017)	Ability of performing the ADL at home, work, or play without undue risk of injury or fatigue.							
Aragao-Santos et al. (2020)	Strength		Jump ability		Gait speed		Quality of life	
La Scala Teixeira et al. (2017)	Strength	Power	Balance	Coordination	Endurance	Speed	Agility	Flexibility

**Table 5:** Neuromuscular adaptations to high-intensity functional training programs.

**References**	**Neuromuscular adaptations to high-intensity functional training**						
Feito et al. (2018)	Strength	Power	Flexibility	Speed	Agility	Endurance	Body composition
Teixeira et al. (2020)	Strength		Power	Speed		Body composition	
Gomes et al. (2020)	Strength	Power	Flexibility	Speed	Agility	Endurance	Body composition
Ben-Zeev and Okun (2021)	Strength	Power	Flexibility	Speed	Agility	Endurance	Body composition

**Table 6:** Neuromuscular adaptations to functional fitness training programs.

**References**	**Neuromuscular adaptations to functional fitness training programs**			
Thompson (2021)	Strength	Power	Balance	Coordination	Endurance
Tibana et al. (2019)	High global performance				

### Exercises Employed in Functional Training Programs

The description of exercises employed in FT programs was extracted from the definitions presented and consulting the training protocols described in the methods section. Tables 7–9 present the FT, HIFT, and FF exercises employed.

**Table 7:** Type of exercises employed in functional training programs.

**References**	**Exercises employed in functional training programs**			
Lajoso-Silva et al. (2021)	Multi-articular movements			
Gali et al. (2021)	Olympic weightlifting	Strength exercises	Plyometrics	Endurance training
McLaughlin et al. (2020)	Functional activities			
Farrokhian et al. (2020)	Daily routine activities	Walking	Climbing up stairs and going down	Getting up and sitting down and move light things
Da Silva-Grigoletto et al. (2020)	Resistance training and associated techniques			
Cheng et al. (2020)	Olympic weightlifting	Gymnastics	Modular movements	
Aragao-Santos et al. (2020)	Squats		Daily routine activities	
La Scala Teixeira et al. (2017)	Strength exercises			

**Table 8:** Type of exercises employed in functional training programs in high-intensity functional training programs.

**References**	**Exercises employed in high-intensity functional training programs**						
Feito et al. (2018)	Functional movements						
Teixeira et al. (2020)	Functional movements	Olympic weightlifting	Running	Rowing	Gymnastics	Constantly varied exercises	
Gomes et al. (2020)	Functional movements	Olympic weightlifting	Running	Rowing	Body weight movements	Squats	Push-ups
Browne et al. (2020)	Functional movements	Cardiovascular exercises			Resistance training		
Ben-Zeev and Okun (2021)	Functional movements				Strength exercises		

**Table 9:** Type of exercises employed in functional fitness training programs.

**References**	**Exercises employed in functional fitness training programs**			
Thompson (2021)	Strength training			
Tibana et al. (2019)	Olympic weightlifting	Gymnastics	Cardiovascular exercises	Sprints

## Discussion

The objective of this study was to investigate whether FT is different from traditional training programs. The main results were: (a) there is no agreement about a universal definition for FT (see Table 1); (b) FT programs aim at inducing the same neuromuscular adaptations to traditional strength, power, and aerobic endurance training programs (see Tables 4–6); (c) exercises employed are also the same (see Tables 7–9). Our main finding is that the FT is not different from traditional strength, power, and aerobic endurance training, therefore, corroborating our hypothesis.

Although muscle strength, power, flexibility, and muscular endurance are well-defined concepts used in exercise prescription (Knuttgen and Komi, 2003; Winter and Fowler, 2009; Cormie et al., 2011a,b; Granata et al., 2018; Suchomel et al., 2018), FT, HIFT and FF problems arise in several domains. Regarding definitions, some studies stated that FT involves resistance training (La Scala Teixeira et al., 2017), and that FF has been defined as a trend toward using strength training (La Scala Teixeira et al., 2017; Thompson, 2021). Thus, both could be easily described as a strength training program. In addition, FT was related to developing different physical capacities in an “integrated and balanced manner” (La Scala Teixeira et al., 2017). Although, strength exercises combined with endurance exercises could be described as “combined,” or “concurrent training.” Therefore, there is no need to “create” new terminology (i.e., FT) containing inconsistencies (Ide et al., 2021).

HIFT was defined as typically involving high-volume and high-intensity exercises, with short rest intervals using multi-joint exercises (Knapik, 2015). This definition consists of a basic description of a strength, power and endurance session adopted as a part of the preparation of elite athletes in specific phases of periodization (Haff and Nimphius, 2012; Haff and Stone, 2015; Suchomel et al., 2018). FF was stated to be also known as HIFT (Tibana et al., 2019). Thus, FF is HIFT, and the difference found between FT and HIFT programs is inconsistent. In addition, since exercise intensity is a training variable and not an exercise type, it would be expected that FT and HIFT were defined as the same training program performed with different intensities only. It was surprising that they are considered separate entities.

Another inconsistency in the FF definition was the use of the expression *extreme conditioning program* (Tibana et al., 2019). If the adjective “*extreme*” is employed to classify these programs, what kind of adjective should we use to classify the physical training programs performed by elite or professional athletes? We are conscious that physical training programs promoted by some fitness companies (e.g., CrossFit^®^, Insanity^®^, Gym Jones^®^, and P90X^®^) were previously classified as *extreme conditioning programs* (Knapik, 2015). They are defined as typically involving high-volume and high-intensity exercises, with short rest intervals and multi-joint exercises (Knapik, 2015). Some include Olympic-style weightlifting and high-intensity interval training, plyometrics, and ballistic exercises (Knapik, 2015). Nevertheless, this training configuration is not exclusive, as it has already been adopted as a part of training programs of elite athletes in specific phases of periodization (Suchomel et al., 2018). Additionally, *High global performance* cited as an objective of FF program (Tibana et al., 2019) represents a highly vague and inconsistent adaptation.

Regarding neuromuscular adaptations, the studies claim that FT programs aim to increase efficiency and safety during activities related to daily living, work, and sports (La Scala Teixeira et al., 2017). Nevertheless, all these benefits are already well-related to the practice of traditional training programs (Cormie et al., 2011a,b; Buchheit and Laursen, 2013; Egan and Zierath, 2013; Baar, 2014; Hughes et al., 2018). Thus, it is not an exclusive or differentiating characteristic of FT programs *per se* (Ide et al., 2021).

Some of the FT problems stated above were previously highlighted (Ide et al., 2021), but we considered them pertinent to highlight again. Muscular fitness is composed of the functional parameters of strength, endurance, and power, and each improves consequence to an appropriately designed resistance training regimen (Garber et al., 2011). The definition of physical fitness implies an optimal combination of physical, physiological, biochemical, biomechanical and psychological characteristics that contribute to competitive success in sports (Shephard, 2000). Physical fitness is specific to competition level (Shephard, 2000) and fitness component (e.g., cardiorespiratory, muscular strength and endurance, body composition, flexibility, and neuromotor fitness) (Garber et al., 2011). Also, the two FF definitions we found (Tibana et al., 2019; Thompson, 2021) do not align with the American College of Sports Medicine position stand (Garber et al., 2011). The ACSM position stand states that FF training incorporates motor skills such as balance, coordination, gait, agility, and proprioception, with physical activities such as tai ji (tai chi), qigong, and yoga (Garber et al., 2011).

The main confusion about all these “new” training programs (i.e., FT, HIFT and FF) is that they often overlap with traditional strength, power, endurance, and flexibility programs (see Tables 4–6). Functional movements/exercises/activities are often cited as training stimuli, but the non-functional movements are not defined. To the best of our knowledge, there is also no concise definition of functional movements as well. By the way, is there any non-functional movement performed by skeletal muscles?

A particular concern was placed in Chodzko-Zajko et al. (2009) study, where gait, balance, and FT were considered as different training interventions. In their study (McLaughlin et al., 2020), an overview of systematic reviews examined the effect of Balance and FT on health outcomes in adults aged 18 years or older. The authors concluded that balance and FT reduced the rate of falls and improved physical function in healthy community-dwelling adults aged 65 years and older (McLaughlin et al., 2020). This separation observed of training interventions to improve gait and balance from FT reinforces FT definitions' confusions, inconsistencies, and weaknesses.

FT, HIFT, and FF training programs present several similarities to those already used for elite athletes for several decades (Chodzko-Zajko et al., 2009; Cormie et al., 2011a; Garber et al., 2011; Haff and Nimphius, 2012; Haff and Stone, 2015). Among them were high-volume and high-intensity exercises, with short rest intervals using multiple joint exercises (Knapik, 2015) and variations of the Olympic-style weightlifting, high-intensity interval training, plyometrics and ballistic exercises (Knapik, 2015). These training parameters are also employed by professional athletes and recommended for developing and maintaining the cardiorespiratory, musculoskeletal, and neuromuscular function of healthy adults and older adults (Chodzko-Zajko et al., 2009; Cormie et al., 2011a; Garber et al., 2011; Haff and Nimphius, 2012; Haff and Stone, 2015). One of the definitions states that FT uses strength exercises aimed at improving core stability (La Scala Teixeira et al., 2017). Curiously, a systematic review concluded that free weight exercises (squat and deadlift) are optimal to achieve this core stability and that abdominal-specific activities or adding balls/devices appear unnecessary (Martuscello et al., 2013). The systematic review results (Martuscello et al., 2013) reinforce that if one of the objectives of FT is to improve core stability, traditional strength and power exercises are the most efficient.

Curiously, one paper provides an equivocal separation of *traditional resistance training* and FT (Da Silva-Grigoletto et al., 2019). *Traditional resistance training* was considered a conservative training method using machines with linear progressive loading (Stenger, 2018). Conversely, FT combined multi-planar, coordinated, and multi-articular movements prescribed via block periodization (Da Silva-Grigoletto et al., 2019). Considering that all these exercises are often employed in athletes' strength and power training, there is no rationality in separating *traditional* from FT programs.

Indeed, the term FT originated in sports medicine and, more specifically, in rehabilitation clinics (Stenger, 2018). Early definitions focused on rehabilitation to enhance or develop the skills associated with activities of daily living and, frequently, involving older adults (Stenger, 2018). In this context, the desired outcome is to restore (or rehabilitate) neuromuscular function. Guidelines and arguments for implementing FT for back pain prevention are essentially the same for back pain rehabilitation (Wirth et al., 2017). This is because the “functional” status of rehabilitation exercises is related to the activities and functions of the body and contextual factors such as environmental and personal factors (World Health Organization, 2013). Although, strength and conditioning professionals are constantly working to improve a specific neuromuscular function. Therefore, the term FT becomes redundant and confusing (Ide et al., 2021).

Fleck and Kraemer (Fleck and Kraemer, 2014) proposed that the general definition of FT is the training that is meant to increase performance in some functional tasks, such as activities of daily living or tests related to athletic performance (Fleck and Kraemer, 2014). Thus, FT could refer to virtually any training meant to increase motor performance (Fleck and Kraemer, 2014). Considering that in exercise physiology, muscle strength, power, flexibility, and endurance are often regarded as functional aspects of the neuromuscular system, this general definition presented by Fleck and Kraemer (Fleck and Kraemer, 2014) appears to be the most rational.

## Conclusions

Exercise adaptations are highly dependent on the specific training stimulus (Nader, 2006; Egan and Zierath, 2013; Hughes et al., 2018). Therefore, an apt description of physical training programs is essential for planning neuromuscular, cardiovascular, metabolic, and functional exercise performance and recovery enhancements. The current study data show that FT has no consistent and universal definition. FT programs and exercises are not different from those already used in sports training, and the claimed neuromuscular adaptations are also the same. In other words: There is no “non-functional” or “traditional training.” Therefore, there is no rationale in classifying exercise training programs as FT. Insisting to use this term (i.e., FT) is a classic case of needlessly reinventing the wheel (Ide et al., 2021). The rational statement is that FT is redundant and should have no place in scientific literature. On the other hand, we agree that, as everyday jargon in practice, the term FT may help coach cues and informal communication between athletes and coaches.

## Future Recommendations

Based on the current results, we recommend that the terms FT, HIFT, and FF no longer describe any physical training program. These can be easily classified as strength, power, endurance, flexibility, and described according to the specific exercises employed (e.g., traditional resistance training, ballistic exercises, continuous and high-intensity interval training).

Sports activities may be broadly classified into events that require great expressions of strength and power (e.g., Olympic-style weightlifting, powerlifting, and throwing events in track and field) and endurance (e.g., marathon run and triathlon) (Nader, 2006). In addition, many activities like middle-distance sprint running and team and combat sports, which are characterized by intermittent efforts, require combinations of high levels of strength and power, combined with a well-developed aerobic capacity for peak performance (Nader, 2006). Table 10 summarizes the skeletal muscle functional proprieties definition and exercises used for their development.

**Table 10:** Skeletal muscle adaptations and exercises employed in strength, power, endurance (aerobic or cardiorespiratory), and flexibility training programs.

	**Definition**	**Exercises used in the training programs**
Strength	The force or torque can be developed by the muscles performing a particular joint movement (e.g., elbow flexion, knee extension) (Knuttgen and Komi, 2003).	→ Traditional resistance training. → Ballistic exercises. → Plyometrics. → Olympic-style weightlifting. → Sprints and resisted sprints.
Power	The rate of performing work; the derivative of work concerning time; the product of force and velocity (Knuttgen and Komi, 2003).	
Endurance (aerobic or cardiorespiratory)	The ability to maintain either a specific isometric force, or a specific power level, involving combinations of concentric and eccentric muscular actions (Winter and Fowler, 2009).	→ Low- and moderate-intensity continuous exercise. → Interval training variations (e.g., HIIT, RST, and SIT).
Flexibility	The intrinsic property of body tissues determines the range of motion achievable without injury (Knudson et al., 2000).	→ Static, dynamic/ballistic stretching. → Proprioceptive neuromuscular facilitation stretching.

*HIIT, High-intensity interval training; RST, repeated sprints training; SIT: sprint interval training*.

In addition to physical training, literature presents several adaptations and health benefits to endurance, strength and power training that may also be used in the proper classification of training stimulus (Egan and Zierath, 2013). Our intention with this article was not to disqualify the studies, physical training programs, and the practice of the physical activities but to provide the correct definitions of terms and concepts to allow proper communication between students, coaches, athletes, and sports scientists.

## Author Contributions

BI conceived the idea, performed the initial data collection, wrote the first draft, worked on all drafts, and formatted the manuscript for submission. AS, MM, CS, BS, DO, and GM helped to develop the main idea and draft the paper. All authors read and approved the last version of the manuscript.

## Conflict of Interest

The authors declare that the research was conducted in the absence of any commercial or financial relationships that could be construed as a potential conflict of interest.

## Publisher's Note

All claims expressed in this article are solely those of the authors and do not necessarily represent those of their affiliated organizations, or those of the publisher, the editors and the reviewers. Any product that may be evaluated in this article, or claim that may be made by its manufacturer, is not guaranteed or endorsed by the publisher.
